# Pathogenic Landscape of Transboundary Zoonotic Diseases in the Mexico–US Border Along the Rio Grande

**DOI:** 10.3389/fpubh.2014.00177

**Published:** 2014-11-17

**Authors:** Maria Dolores Esteve-Gassent, Adalberto A. Pérez de León, Dora Romero-Salas, Teresa P. Feria-Arroyo, Ramiro Patino, Ivan Castro-Arellano, Guadalupe Gordillo-Pérez, Allan Auclair, John Goolsby, Roger Ivan Rodriguez-Vivas, Jose Guillermo Estrada-Franco

**Affiliations:** ^1^Department of Veterinary Pathobiology, College of Veterinary Medicine and Biomedical Sciences, Texas A&M University, College Station, TX, USA; ^2^USDA-ARS Knipling-Bushland U.S. Livestock Insects Research Laboratory, Kerrville, TX, USA; ^3^Facultad de Medicina Veterinaria y Zootecnia, Universidad Veracruzana, Veracruz, México; ^4^Department of Biology, University of Texas-Pan American, Edinburg, TX, USA; ^5^Department of Biology, College of Science and Engineering, Texas State University, San Marcos, TX, USA; ^6^Unidad de Investigación en Enfermedades Infecciosas, Centro Médico Nacional SXXI, IMSS, Distrito Federal, México; ^7^Environmental Risk Analysis Systems, Policy and Program Development, Animal and Plant Health Inspection Service, United States Department of Agriculture, Riverdale, MD, USA; ^8^Cattle Fever Tick Research Laboratory, United States Department of Agriculture, Agricultural Research Service, Edinburg, TX, USA; ^9^Facultad de Medicina Veterinaria y Zootecnia, Cuerpo Académico de Salud Animal, Universidad Autónoma de Yucatán, Mérida, México; ^10^Facultad de Medicina Veterinaria Zootecnia, Centro de Investigaciones y Estudios Avanzados en Salud Animal, Universidad Autónoma del Estado de México, Toluca, México

**Keywords:** Lyme borreliosis, VEE, Hantavirus, *Babesia*, Chagas, *Leishmania*, pathogenic landscapes, global change

## Abstract

Transboundary zoonotic diseases, several of which are vector borne, can maintain a dynamic focus and have pathogens circulating in geographic regions encircling multiple geopolitical boundaries. Global change is intensifying transboundary problems, including the spatial variation of the risk and incidence of zoonotic diseases. The complexity of these challenges can be greater in areas where rivers delineate international boundaries and encompass transitions between ecozones. The Rio Grande serves as a natural border between the US State of Texas and the Mexican States of Chihuahua, Coahuila, Nuevo León, and Tamaulipas. Not only do millions of people live in this transboundary region, but also a substantial amount of goods and people pass through it everyday. Moreover, it occurs over a region that functions as a corridor for animal migrations, and thus links the Neotropic and Nearctic biogeographic zones, with the latter being a known foci of zoonotic diseases. However, the pathogenic landscape of important zoonotic diseases in the south Texas–Mexico transboundary region remains to be fully understood. An international perspective on the interplay between disease systems, ecosystem processes, land use, and human behaviors is applied here to analyze landscape and spatial features of Venezuelan equine encephalitis, Hantavirus disease, Lyme Borreliosis, Leptospirosis, Bartonellosis, Chagas disease, human Babesiosis, and Leishmaniasis. Surveillance systems following the One Health approach with a regional perspective will help identifying opportunities to mitigate the health burden of those diseases on human and animal populations. It is proposed that the Mexico–US border along the Rio Grande region be viewed as a continuum landscape where zoonotic pathogens circulate regardless of national borders.

## Introduction

The United States (US) and Mexico share a border spanning 3,100 km from the Gulf of Mexico to the Pacific Ocean. Approximately 14 million people reside within the area found roughly 100 km on either side of the international line between the two countries, with 7.3 million residing in the US and 6.8 million in Mexico (1). A bi-national effort is in place to protect the environment and public health in the US–Mexico border region that is consistent with sustainable development. The sector encompassing the border States of Texas, Tamaulipas, Nuevo León, and Coahuila includes at least 29 municipalities on the Mexican side and 168 cities and towns on the US side, covers portions of the Southern Texas Plains and Western Gulf Coastal Plain ecoregions of Texas, and lies within a zoonotic disease hotspot (1–3). It appears that conditions leading to the emergence of zoonotic diseases as a public health threat in the US and other parts of the world may be at play in the transboundary region covering south Texas and Northeast Mexico (4–7). Among these factors, we have poverty. For instance, in southern US, 16.5% of the population is in poverty, and 22% of children under 18 years old live in such conditions in the same region [National Center for children in poverty^1^ (8)]. Texas has a poverty level (17.6%) higher than the national average (15%) calculated as a 3-year average. In addition, recent studies showed that migrants displaced due to adverse weather conditions related to climate change, are predicted to increase during the twenty-first century (9, 10). Economically, deficient areas will be highly impacted with these types of extreme weather events, making this population more vulnerable to emerging infectious diseases due to an increase in out-migration flow (9).

These scenarios acquire special relevance in the US–Mexico border, one the largest and longest-sustained routes of human migration. From the human migratory standpoint, the States of Chiapas and Tabasco in Mexico play a vital role as transit points for large numbers of people. In this regard, the ports of entry are the Ocosingo and Tapachula surroundings in Chiapas, and Tenosique in Tabasco. Veracruz is therefore the transition point for the nearly 400,000 individuals, representing approximately 50 nationalities, who traverse Mexico each year with the ultimate goal of reaching the US (Migration Department, Mexican Interior Ministry 2006). All these individuals are forced to cross areas that might be “hot spots” of Babesiosis, Venezuelan equine encephalitis virus (VEEV), and other pathogens that have incubation periods fluctuating between 3 and 10 days (3). Consequently, the “One Health” approach is required to enhance the ability to recognize zoonotic pathogens in humans, domestic, and wildlife reservoirs and the associated vectors in the US and Mexico transboundary region. This concept states simply that clinicians, researchers, agencies, and governments must work together seamlessly for the benefit of animal and human health as well as for the welfare of the global environment.

An international perspective on the interplay between disease systems, ecosystem processes, land use, and human behaviors is applied in this review paper to analyze landscape and spatial features of Venezuelan equine encephalitis (VEE), Hantavirus disease, Lyme disease (LD), Leptospirosis, Bartonellosis, Chagas disease, Babesiosis, and Leishmaniasis all of which can be considered, or have the potential to be emerging zoonotic infectious diseases of relevance in this transboundary region (Table 1) (11–15).

**Table 1:** **Transboundary zoonotic diseases, distribution, agents, vectors, and transboundary relevance in the US–México border region**.

Disease	Distribution^a^	Etiologic agent	Vector	Transboundary relevance
**VIRUS**				
VEE	Meso-America, Southern Texas, and Northern Mexico	Venezuelan equine encephalitis virus	*Culex (Melanoconion) taeniopus*	Shared vectors and reservoirs
			*Deinocerites pseudes*	Human migration
			*Aedes (Ochlerotatus) taeniorhynchus*	Livestock movement
			Mammalophilic mosquitoes	
HPS and HFRS	American continent, Europe, Asia, Africa likely worldwide	Hantavirus	Wild rodents of the Cricetidae and Soricidae families serve as reservoirs	Shared reservoir species across border
				Human migration
				Different public health preparedness
				Poverty (suboptimal housing)
**BACTERIA**				
Lyme disease	US, Mexico, Canada	*Borrelia burgdorferi*	Ticks	Shared vectors and reservoirs
			*Ixodes scapularis*	Different public health policies
			*I. pacificus*	
Leptospirosis	Worldwide	*Leptospira interrogans*	Wild rodents serve as reservoirs	Shared reservoir species across borders
				Human migration
				Livestock movement
				Different public health policies
				Poverty (suboptimal housing, sanitation, and hygiene)
Rocky mountain spotted fever/Brazil spotted fever	US, Mexico, Canada, Costa Rica, Panama, Colombia, Uruguay, Argentina, Brasil	*Rickettsia rickettssi*	Ticks	Shared vectors and reservoirs
			*Dermacentor variabilis*	
			*D. andersoni*	
			*Rhipicephalus sanguineus*	
			*Amblyomma cajennense*	
			*Haemaphysalis leporispalustris*	
Human monocytic ehrlichiosis	US, Mexico	*Ehrlichia chaffensis*	Ticks	Shared vectors and reservoirs
		*E. ewingii*^e^	*A. americanum*	
			*D. variabilis*	
			*I. pacificus*	
Human granulocytic anaplasmosis	US	*Anaplasma phagocytophilum*	Ticks	Shared vectors and reservoirs
			*I. scapularis*	
			*I. pacificus*	
Bartonellosis^b^	Americas, Europe, Asia	*Bartonella henselae*	Ticks	Shared vectors and reservoirs
		*Ba. quintana*	*I. pacificus*	Human migration
		*Ba. bacilliformi*	*I. scapularis*	Livestock movement
		*Ba. vinsonii* subsp. *berkhoffii*	*I. ricinus*	Poverty (poor sanitation, hygiene and crowded housing environments)
			*I. persulcatus*	
			*D. reticulatus*	
			*D. marginatus*	
			*R. sanguineus*	
			*R. microplus*	
			Sand flies	
			*Lutzomyia verrucarum*	
			*L. columbiana*	
			*L. peruensis*	
			Lice^c^	
			*Pediculus humanus humanus*	
			*P. capitis*	
			Fleas^c^	
			*Pulex irritans*	
**PROTOZOA**				
Human Babesiosis	US	*Babesia microti*	Ticks	Shared vectors and reservoirs
			*I. scapularis*	
			*I. pacificus*	
			*I. texanus*	
			*D. variabilis*	
Chagas^d^	American Continent	*Trypanosoma cruzi*	*Triatoma sanguisuga*	Shared vectors and reservoirs
			*T. gerstaeckeri*	Human migration
			*T. lenticularia*	Different public health policies
				Poverty (suboptimal housing)
Leishmaniasis	Americas	*Leishmania (Leishmania)*	*Lutzomyia* sand flies	Shared vectors and reservoirs Human migration Different public health policies
		*Leishmania (Viannia)*		

*^a^Adapted from Ref. (173)*.

*^b^With relevance to this review paper*.

*^c^Human body and head lice, and human fleas. Other species have been associated with wildlife and domestic animals (173)*.

*^d^As per Ref. (298, 304)*.

*^e^In the US only*.

## Pathogenic Landscape

### Global change and emergence of vector-borne zoonotic diseases

As the world globalizes in terms of Nation’s economies and increased travel, borders are opened for a constant flow of goods, products, and pathogen dissemination. Likewise, as human populations expand into new geographical regions, the possibility that humans will come into close contact with infectious agents’ potential hosts, which can transmit pathogens to human beings, increases. Such factors, combined with increased human density and mobility, stand as a serious human health threat. Additionally, climate change is increasingly becoming a concern in the emergence of zoonotic infectious diseases (16). For the past 70 years, most of the newly emergent diseases have been identified as zoonoses (60.3% of EIDs), and the majority (71.8%) has originated in wildlife (17). Thus, according to the transboundary zoonotic disease concept, understanding how these pathogens emerge in different geographical regions will directly benefit global trade and public health. Here, we review several diseases that could impact a geographically strategic region in the US and Mexico border area. Insights gained understanding the pathogenic landscape of these zoonotic diseases could help enhance predictive tools, which might be applied to study the epidemiology of other transboundary pathogens. Pathogenic landscape is a term used to describe attributes of an ecosystem that influence spatial variations in disease risk or incidence (18).

Emission of greenhouse effect gasses has impacted global climate (19), increasing Earth’s surface temperature 0.74°C on average (20). According to the 2002 World Health Organization (WHO) report, climate change has caused approximately five million disability-adjusted life years (DALYs) in the world (21). Moreover, the 2007 Intergovernmental Panel on Climate Change report (22) and the 2014 National Climate Assessment by the US Global Research Program (16), suggest that climate change will affect North America at multiple levels, such as public health, agriculture, water supply, and frequency of extreme weather events, among others (15). Increased temperatures, sea levels, precipitations, and droughts due to climate change can drastically change the epidemiology of vector-borne diseases (15, 21, 23, 24), as both vectors and pathogens are very sensitive to these climatic variables.

Climate change can potentially alter the spatial range of vector-borne diseases through shifts in geographical distributions of their vectors (14, 25, 26). Despite some positive developments, such as better access to clean drinking water, lower exposure to insect vectors, and higher-quality housing, projected changes in climate over the next decades may exacerbate infectious disease incidence even in developed regions such as North America (15). Habitat changes, alterations in water storage and irrigation habits, pollution, development of insecticide and drug resistance, globalization, tourism, and travel are additional factors that may aggravate this threat (26). For instance, in Europe, short winters appear to have influenced populations of *Dermacentor reticulatus*, the tick vector responsible for transmission of *Babesia canis*, to expand to the East (20). In Veracruz, Mexico, a study found an association between an increase in dengue cases and increased temperature and rainfall that followed El Niño Southern Oscillation (ENSO) events (27). Increased rainfall could create both microclimates, in which vectors can thrive, as well as cause high temperatures, which could allow for a rapid increase in vector densities and ultimately put humans at risk for vector-borne disease. Extreme-flooding events can cause outbursts of zoonotic diseases caused by infectious agents transmitted by rodents, as their pathogen-containing urine contaminates the water. This was the case in Nicaragua, in which a Leptospirosis epidemic followed a flooding event (28). Thus, humans might face increasing exposure to zoonotic diseases as naturally occurring phenomena like ENSO and flooding events are expected to become frequent due to climate change (23). In addition, climate change can also affect the epidemiology of zoonoses indirectly. For instance, the density of vegetation in a particular area increases during heavy rainfall seasons. This vegetation indirectly supports the reproduction of rodents, which can be infected with pathogens transmissible to humans (29).

Human migration and economic trade can exacerbate climate change influences on vector-borne diseases along the Texas–Mexico border. Ecological niche models, under climate change scenarios, showed an increased distribution of Leishmaniasis vectors and reservoirs in Texas and North Mexico (14). A recent study in the Texas–Mexico border identified the present and future potential distribution, under climate change scenarios, of the LD vector, *Ixodes scapularis* (13). The results of this study indicated that South Texas includes suitable habitat for *I. scapularis*. In a similar study, the potential future distribution of main Chagas disease vectors, *Triatoma gerstaekeri* and *T. sanguisuga*, is expected to increase in the Texas–Mexico border due to climate change (12). Temperature and precipitation played a major role in the models presented in these three studies.

The transboundary region between Mexico and the US is vulnerable to outbreaks of vector-borne diseases because some Southern States, such as Texas, share a legacy of neglected tropical diseases (NTD) (30) with Mexico. This situation highlights the urgency to develop and deploy active surveillance programs, which are necessary for optimal management and control of vector-borne diseases.

### Effect of exotic weeds on vector populations

**Figure 1:**
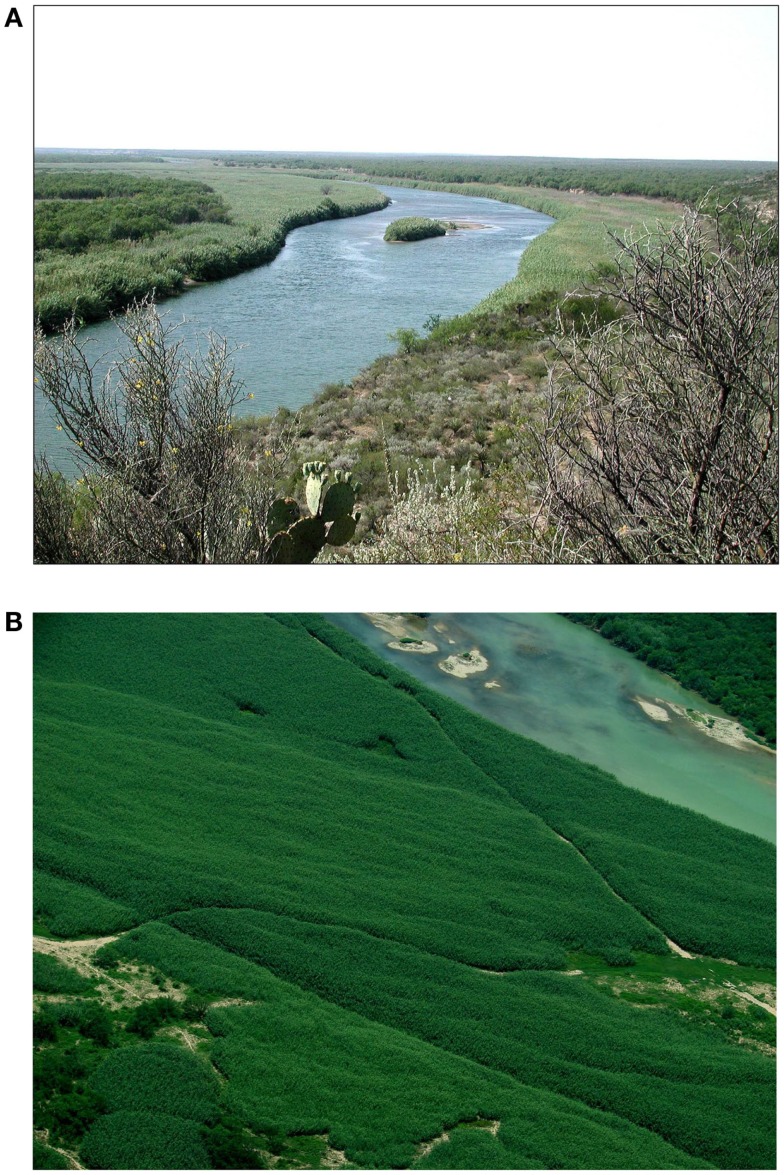
**Invasion of exotic *Arundo donax*, giant reed, is facilitating the invasion of Cattle Fever Ticks in the transboundary region by creating a microclimate that is favorable for its survival**. **(A)** *Arundo donax* on Rio Grande near Eagle Pass, TX, USA. **(B)** *Arundo donax* on Rio Grande near Del Rio, TX, USA.

Invasive weeds can change the ecology of, and induce a pathogenic landscape in which arthropod-borne disease transmission can increase. Mack and Smith (31) link invasive plants as catalysts for the spread of human parasites by documenting the escape and mast seed production of Asian frost-tolerant bamboos from cultivation in the Pacific Northwest to potential outbreaks of the omnivorous deer mouse *Peromyscus maniculatus* that carries Hantavirus. Invasive weeds also interact with ticks. Japanese barberry has been shown to increase the abundance of the blacklegged tick, *I. scapularis* and the infection prevalence of *Borrelia burgdorferi* (LD) (32, 33). In India, Kyasanur forest disease of cattle and monkeys is attributed to disturbance of the native forest for tea plantations, which resulted in invasions of the invasive weed *Lantana camara* and outbreaks of *Haemaphysalis spiniger*, the vector for Kyasanur encephalitis-inducing virus within the *flavivirus* group (34). The clearing of the native forest in Argentina and the transition to exotic African grasses increased the impact of cattle fever ticks (CFT) by increasing the encounter rate with cattle (35). This same pathogenic landscape phenomenon appears to be happening in the permanent quarantine zone (PQZ) in the Texas–Mexico border region, with the invasion of the exotic and invasive weed species, *Arundo donax* known as the giant reed (Figure 1). Aerial remote sensing pictures of the Rio Grande taken in 2002 indicate that 62% or 5981 ha of riparian habitat on Rio Grande from Big Bend to Falcon Dam was infested with giant reed, which includes most of the PQZ (36). Zoonotic agents are not transmitted by CFT, but knowledge from the study of CFT ecology can be applied to understand how ecosystem shifts can influence spatial variation in disease risk or incidence for tick-borne disease systems of public health importance.

Giant reed impacts the USDA-APHIS Cattle Fever Tick Eradication Program (CFTEP) along the transboundary region of Texas–Mexico border. Giant reed indirectly affects CFT because survival is highest in giant reed as compared to native riparian vegetation or buffel grass pastures (37). Abiotic conditions within giant reed stands are cooler due to the heavy shade and high rates of evapotranspiration (ET), which appears to be cause of lower levels of CFT mortality. Biotic conditions in giant reed stands are also altered because the abundance of CFT arthropod predator species is reduced (37). In a review of the literature records of predation on ticks, Samish & Alekseev (38) documented that ground dwelling predators (e.g., ants, beetles, and spiders) are the major natural enemies of ticks. Preliminary pitfall trap surveys in the PQZ indicate that ground dwelling beetle populations, specifically the predaceous Carabidae and omnivorous Tenebrionidae species, are strongly reduced in giant reed compared to adjacent native plant communities (Goolsby, personal communication). Ants are also known to be important predators of ticks in Texas. Fleetwood et al. (39) documented reduced populations of Lone Star ticks, *Amblyomma americanum*, in pastures with abundant red imported fire ants, *Solenopsis invicta*. Fire ant predation is generally believed to reduce the incidence of tick-vectored pathogens of livestock. In Louisiana, fire ant predation of *Ixodes* ticks was associated with a reduced incidence of Anaplasmosis in cattle (40). Preliminary studies in the PQZ found that ant diversity and abundance is low in giant reed stands, with the red imported fire ant, *Solenopsis invicta*, being the most common species. Comparative studies are needed to survey ant diversity throughout the PQZ to investigate their potential impact on CFT. Control of exotic giant reed and restoration of the native riparian vegetation could reduce the favorability of this habitat for CFT and lead to restoration of a more intact leaf litter insect predator community and in total a more robust biological barrier to invading CFT and other tick-borne zoonoses. Giant reed may also be creating a localized climatic refuge for CFT when conditions in the upland habitat are not favorable for survival. As giant reed declines, lower ET rates, increased ground temperatures, and lower humidity levels are expected in these riparian habitats and these conditions are known to reduce the survival of larval and adult CFT (41–45). Giant reed also indirectly impacts the CFTEP by reducing visibility in the PQZ along the Rio Grande. Heavy infestations of giant reed make it extremely difficult for mounted inspectors to detect and capture stray livestock.

Exotic weeds interact with disease vectors in the transboundary region between Texas and Mexico. These weeds create a landscape that is depauperate of beneficial predators of disease vectors and alter the microclimate, and as such, they can facilitate the invasion of these vectors and must be considered in their full ecological context.

## Zoonotic Infectious Diseases

### Viral infectious diseases

#### Venezuelan equine encephalitis

Venezuelan equine encephalitis viruses are members of the VEE complex and comprise the three major serogroups of New World alphaviruses (46, 47). Fourteen subtypes and varieties have been described within the VEEV complex (48). The IAB and IC viruses are designated “epidemic” or “epizootic” because they have been isolated only during equine and human outbreaks. They are distinct from enzootic strains (subtypes/varieties ID-F, II-VI) that circulate in sylvatic or swamp habitats, and occasionally cause disease in humans or domestic animals (48–50). Importantly, VEEV isolates identified to be of the IE subtype identified during epizootics in Mexico appear to be equine neurovirulent, but are unknown to produce high titer viremia (50). Transmission cycles have been described for most of the enzootic VEE subtypes/varieties [ID, IE, II, IIIA, and IIIB (Tonate)], except III C and V (48). Most of them are transmitted among rodents by mosquitoes in the subgenus *Culex* (*Melanoconion*) and few mammalophilic mosquitoes (51).

Venezuelan equine encephalitis virus was known to be circulating and producing illness in horses since the 1920s (49, 51), and in 1938 the virus was isolated from the brain of a horse that died of encephalitis in South America (52–54). Human cases with neurological complications were recorded in 1950 during an outbreak of febrile illness in Espinal, Colombia (55). VEEV outbreaks continued at regular intervals through the 1960s in South America affecting tens-to hundreds-of-thousands of people (50, 56). Between 1973 and 1992 no VEEV was documented, which led to the assumption that the epidemic-epizootic subtypes IAB and IC VEEV had disappeared (50). However, phylogenetic studies and renewed epidemic/epizootic VEE activity in Northwestern Venezuela during 1992–1993 revealed that these viruses remain a threat (57, 58). Two equine epizootics in the States of Chiapas and Oaxaca, Mexico caused by a subtype IE virus (59), and a major outbreak in Venezuela and Colombia during 1995 affecting about 100,000 people (60, 61) draw attention to the continued threat of VEEV. To date, VEEV viruses affecting humans and equids have been found in at least 12 countries of the Americas causing important social and economic damage mainly in Latin America (48).

Most VEEV epizootics and epidemics have taken place in Northern South America and in regions of Venezuela and Colombia. However, VEE has also affected North America on several occasions. In 1966, an equine epizootic in Northeastern Mexico was reported in southern Tamaulipas and Northern Veracruz within the Panuco river basin (62). Although no viruses were isolated, VEE etiology was determined serologically. The lack of VEE vaccination in Mexico at this time suggests that this outbreak was caused by a local enzootic subtype IE strain. Another major Middle American outbreak and one of the most devastating VEEV pandemics of the Continent, began near the Guatemala-El Salvador border on the Pacific coast and spread through much of Central America, Mexico, and Texas during 1969–1972, and involved tens-of-thousands of equines and people. In Mexico, equine deaths from VEE were first reported in 1969 in mountainous regions of the State of Chiapas close to the Guatemalan border (63). By 1970, the epizootic had produced about 10,000 equine deaths in the States of Chiapas and Oaxaca (64). The outbreak then spread northward to the Gulf Coast and eventually reached Southern Texas. In Texas, approximately 1500 horses died of VEE, and several hundred human infections were documented. The Texas outbreak was halted by a massive equine vaccination program and aerial insecticide spraying costing about 15 million dollars (50, 65).

In the 1990s, two outbreaks of equine encephalitis occurred on the Pacific Coast of Southern Mexico. From June to July in 1993, 125 equine cases including 63 deaths were reported in Chiapas State. Three years later, from June to July in 1996, another equine epizootic occurred nearby in Oaxaca State, involving 32 horses with 12 deaths (59, 66). Epidemiological and serological data were consistent with VEE, and VEEV was isolated from encephalitic horses involved in each outbreak. No human cases were documented during these outbreaks. However, further serosurveys and VEEV isolations obtained in the same area of the 1990s outbreaks demonstrated that VEE has been endemic in this Southern region of Mexico for decades (67).

Two virus isolates from the Mexican outbreaks of the 90s were examined antigenically and genetically. All were VEE subtype IE by IFA and ELISA using monoclonal antibodies (68). Sequencing and phylogenetic studies indicated that the outbreak strains belong to one of three major subtype IE VEEV lineages. This lineage circulates on the Pacific Coast of Guatemala, and was sampled there from 1968 to 1980 (59, 66). The absence of previous Mexican isolates from this lineage suggests that the currently circulating Mexican strains originated from enzootic transmission foci on the Pacific Mexican Coast. The remaining IE lineages circulate on the Gulf and Caribbean Coasts of Central America (Nicaragua northward to the Gulf Coast of Mexico and close to the US border) and in Western Panama, and differ by up to 7% at the nucleotide sequence level.

Before the Chiapas–Oaxaca outbreaks, isolates of enzootic VEEV, including subtype IE, were traditionally believed to be avirulent for equines, and were not previously known to have epizootic potential (69–71). Experimental infection of horses with several IE strains from Mexico and Nicaragua showed that these viruses generally produced little viremia and disease (70, 71). Further studies using reverse genetics approaches demonstrated that *Aedes* (*Ochlerotatus*) *taeniorhynchus*, an abundant epizootic vector in coastal areas of Chiapas and Oaxaca, was more susceptible to isolates obtained during the 1993 and 1996 epizootics compared with closely related enzootic IE strains isolated previously in Guatemala (72). A mechanism of VEEV emergence was suggested showing that a single Ser → Asn amino acid substitution at position 218 of the E2 envelope glycoprotein was the major determinant of the increased *Ae. taeniorhynchus* infectivity. Viral adaptation to a vector that prefers to bite large mammals was suggested as the emergence mechanism in the 1990s outbreaks of Southern Mexico (72).

Subtype IE enzootic viruses are the only VEEV known to continuously circulate in Mexico both currently as well as prior to 1993 outbreak. They occur from Western Panama through Tamaulipas State in Mexico (48, 65, 73–75). The ecology of the IE viruses has been studied in detail and *Culex* (*Melanoconion*) *taeniopus* is the known primary enzootic vector of subtype IE viruses in Guatemala (76), and one of the principal vectors maintaining endemic VEEV cycles in southern Mexico, the Gulf Coast of Mexico (48, 73, 75), and Panama (65). It has been demonstrated that *Cx. taeniopus* found in estuarine areas of Chiapas is susceptible to both subtype IE VEEV isolates from the Pacific Mexican outbreaks of the 90s and to strains isolated from hamster sentinels during the 2000s in the same region (77). *Cx. taeniopus* feeds on a wide variety of hosts, mainly small rodents from the Cricetidae family such as cotton rats and rice rats, and seems to circulate subtype IE VEEV not only in the Mexican Pacific Coast but also in the Gulf Coast of Mexico (73, 77). The scenario in the Pacific Coast of Southern Mexico appears to involve VEEV transmission by *Ae. taeniorhynchus* to equids and possibly humans at inland locations (48). The threat of VEEV outbreaks in the Mexican Pacific region involving enzootic and epizootic vectors exists (48). Such region is linked to the East through Gulf river basins where several endemic VEEV foci are found, along the States of Tabasco, Veracruz, and Tamaulipas. The later of these States is adjacent to the US Texas border, and is a spot of subtype IE VEEV activity (73). Hotspot activity was characterized through serosurveys suggesting VEEV infections in cattle, equines, rodents, and humans, which was complemented with the isolation of IE VEEV (73). Findings of the study showed that at least one major urban region (Minatitlan in the State of Veracruz) has active enzootic VEEV transmission with *Cx. taeniopus* identified as the main VEEV vector (74; Estrada-Franco and Weaver, unpublished information). Several mosquito epizootic vectors of VEEV that were found infected and active during the VEEV pandemic of the 1970s are present on both sides of the border (Tamaulipas and Texas), such as *Ae. sollicitans*, *Psorophora confinnis*, and *Ae. taeniorhynchus* (63, 78). The risk of *Ae. taeniorhynchus* or other epizootic vectors adapting to endemic VEEV cycles elsewhere outside the Chiapas area, as was demonstrated with the IE VEEV strains isolated in the Pacific coast, could be of veterinary and public health significance. An epidemic strain of VEEV has the potential to arise from circulating endemic strains, which may be easily misdiagnosed for another febrile-causing disease if appropriate diagnostic assays are not routinely performed. The movement of epidemics by viremic individuals is a major concern, particularly in the Gulf Coast region of Mexico, which could threaten previously unaffected areas of Mexico and even the US. Moreover, direct human-to-human transmission of VEEV has also been suggested by the sudden appearance, rapid increase, and brief occurrence of human disease within affected communities (61, 79).

Reconstructing the historical incidence of VEE could facilitate the forecast of recurring patterns and help improve strategies for disease prevention, e.g., vaccine distribution logistics. Our hypothesis is that drivers of VEE outbreaks are responsive to heavy rainfall events but activity subsides as drier conditions return.

**Figure 2:**
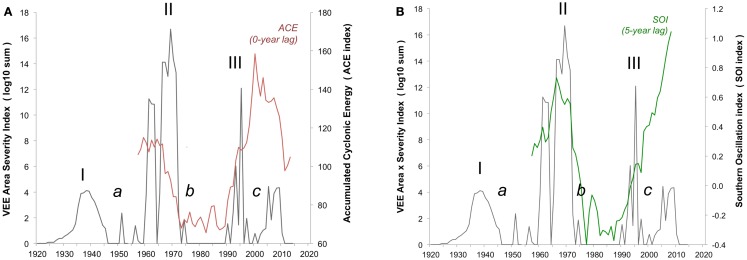
**Integrated VEE area-severity index**. Integrated VEE area-severity index (*dark solid line*, left axis) based on historical reconstruction of areal extent and severity in equine and human populations over the 1920 through 2013 period. Three major VEE outbreaks (I, II, III) and three gap-intervals (*a*, *b*, *c*) of low VEE are shown. **(A)** Eleven-year running means of accumulated cyclonic energy index (ACE, *red font*) and **(B)** the Southern Oscillation Index (SOI, *green font*) given on right axis.

The historical time-series shows three repeating VEE outbreaks over the past century, recurring at approximately 30-year intervals, and spanning up to one decade. The gap of two decades following each outbreak period is conspicuous (Figure 2). Attention to details of each major outbreak show striking differences between epidemics. The first event (Outbreak I, 1935–1946) is exclusively an equine outbreak, with a locus in Columbia and Venezuela. By 1942, it had spread to Peru in the South and by 1943 to Trinidad in the East. The actual number of equines affected in this outbreak was not documented, and there are no records of human VEE cases; the latter do not appear as recorded observations until the 1960-decade. The gaps over the 1970–1980 decades (Gap b) and over the 1996–2013 interval (Gap c) contrast, at least in terms of recent serological evidence, and in terms of important climatic conditions.

The ocean indicators both show low levels of storm impact over the 1972–1992 (Gap b), and likely reflect the paucity of extreme rainfall events, which is in contrast with the high storm levels over the 1996–2013 interval (Gap c) (Figure 2). The latter suggests hurricane and ENSO events are now opportune to bring heavy rainfall into the VEE-affected region. This begs the question of why recent VEE levels remain relatively low and points to the need for further analysis of local rainfall and other weather events across the region.

**Figure 3:**
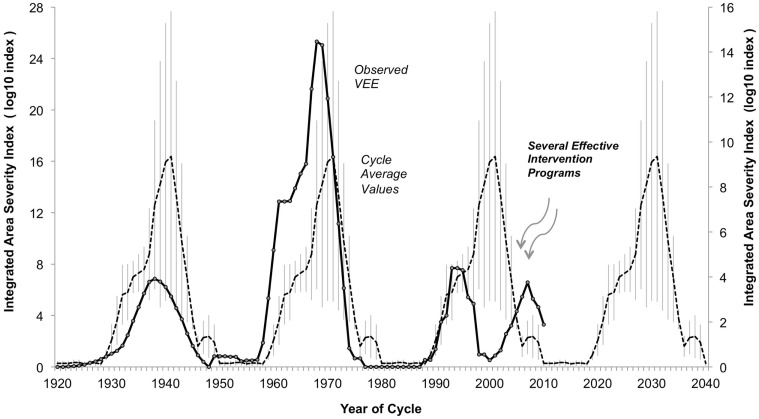
**Observed integrated area-severity index for Venezuelan equine encephalitis (VEE) in the Americas, 1920–2010**. Integrated Area-Severity (IAS) index (*dark solid line*, left axis, log10 scale, 5-year point-centered moving average) for Venezuelan Equine Encephalitis (VEE) in the Americas, based on reconstruction of actual disease incidence reported in literature, 1920–2010. Hypothetical reconstruction of expected levels of VEE, 1920–2010, achieved by sequential repeat of the average IAS index levels over Outbreak Two (1950–1979) and Outbreak Three (1980–2009) (*dashed line*, right axis, log10 scale), with standard error of mean added (*light vertical lines*). Outbreak One (1920–1949) values excluded from average due to lack of solid data. Subdued levels of VEE over Outbreak Three could be the result of assertive mosquito control programs related to dengue fever epidemics, and/or improved VEE management approaches and infrastructure (see text for details).

There is uncertainty and considerable concern over the possible re-emergence of an outbreak of VEE. Our time-series model and its correlation with broad climate signals (Figure 2) offers a new and evocative look at VEE at the century and cross-regional scales. It opens a window on a conceptual framework for better understanding and managing outbreaks of the virus through both the tracking of accelerated climate change (e.g., satellite imagery), and use of long-term forecasting (Figure 3). Work is in progress using power spectrum analysis to quantify likely timing and magnitude of future outbreaks. This is important now that serological analyses show the widespread presence of the VEE virus in humans, equines, and populations of virus reservoirs like the rodent species mentioned above, which mosquito vectors feed on, that respond to unusually strong rainfall events.

The development and persistence of high Accumulated Cyclonic Energy Index (ACE) and equatorial Pacific Ocean Southern Oscillation Index (SOI) levels imply VEE outbreak activity currently and in the near future (Figure 2). Why VEE outbreak activity has remained low since 1966, when broad oceanic indicators suggest a high potential for outbreak, remains unknown, which indicates the need for further research. For example, additional research into local rainfall conditions could help understand how mosquito populations expand, which may drive VEEV activity as it has been shown in other mosquito-borne arboviral disease systems (80). Conditions since the 1972–1992 gap have changed, moving toward better virus monitoring and disease management approaches. These changes, taken with the movement of human populations into cities and a relative decline in equine populations, will also play a role in decreasing the likelihood or severity of a VEE outbreak. There is currently widespread mosquito control in response to dengue epidemics. Any of these developments may be limiting the magnitude of current and near-term VEE epidemics (Figure 3). Outbreaks in the 1930s and 1960s were weakly constrained, at best, and may offer a study in contrast to modern conditions with better knowledge and disease management infrastructure.

#### Hantavirus

The genus *Hantavirus*, from the family Bunyaviridae, is composed of viruses with a three-segment negative sense RNA genome (81, 82). In nature, these viruses are hosted by a variety of rodent and soricomorph species as persistent infections (81). Humans acquire *Hantavirus* infection by inhaling aerosolized particles from rodent excreta and urine, or via the bite of an infected animal (81). Some rodent-borne Hantaviruses are associated with two types of human disease, differing between the New and Old Worlds. In the Americas, *Hantavirus* pulmonary syndrome (HPS) has reached fatality rates of 60% and is characterized by elevated pyrexia, pulmonary dysfunction, and cardiac shock (83). In several regions of the Old World (Europe, Russia, China and Korea), hantaviral infections cause a hemorrhagic fever with renal syndrome (HFRS) characterized by high fever, renal dysfunction, and hemorrhage but with mortality rates usually lower than 12% (81, 83).

A relevant feature of Hantaviruses is their close association between a specific *Hantavirus* and its rodent host species, suggesting a strong relationship between Hantaviruses and their reservoirs (84, 85). Globally, these viruses occur in close association with rodent and shrews of the families Cricetidae and Soricidae, with a majority of New World Hantaviruses detected in reservoir rodent species of the sub-family Neotominae (82, 86). Throughout America, more than 30 Hantaviruses have been identified, but only a few have been associated to HPS (81). The New World geographic distribution of known hantaviral strains includes Canada, US, Mexico, Honduras, Costa Rica, Panama, Venezuela, Peru, Bolivia, Brazil, Argentina, Chile, and Paraguay (81, 83). Rodents with *Hantavirus* antibodies have been detected in Peru, Venezuela, Costa Rica, Honduras, and Mexico, although HPS in humans has not been documented in these countries (81, 87). The distribution of recorded cases of HPS and the distribution of *Hantavirus* seroprevalent rodents in the Americas is not coincidental for several reasons: (1) not all Hantaviruses have been associated with HPS; (2) our knowledge of hantaviral diversity present in the continent is likely incomplete; and (3) HPS may be confused with clinically similar diseases. In North America, five Hantaviruses known to cause HPS are the Sin Nombre virus (SNV), New York virus (NYV), Choclo virus (CHOV), Black Creek Canal virus (BCCV), and Bayou virus (BAYV) (83, 88). SNV is the major cause of HPS in the US and Canada, where *Peromyscus maniculatus* (deer mouse) is the primary rodent reservoir (81, 89).

Northeastern Mexico (Chihuahua, Coahuila, Nuevo León, and Tamaulipas) and Texas share a common biogeographic history, and thus a large number (23) of Cricetinae rodent species are shared among these States (90, 91). Because the abiotic environment and rodent assemblages of Northeastern Mexico are similar to those of areas in the adjacent US, it is likely that many viruses circulating in this region occur on both sides of the international border. However, specific information regarding the prevalence and spatial distribution of Hantaviruses within this region is scarce and incomplete. *Hantavirus* antibody-positive rodents from seven species have been found in Chihuahua, Nuevo León, and Tamaulipas. The only *Hantavirus* identified to date within these species is the SNV. However, because most of the rodent individuals tested were only analyzed by serology tests, it is possible that other Hantaviruses occur in Northeastern Mexico (88, 92, 93). In Texas, antibodies for Hantaviruses have been detected in 11 rodent species and 4 Hantaviruses (SNV; El Moro Canyon virus, ELMCV; Muleshoe virus, MULV; and BAYV) are known to occur in this state (94). No cases of HPS are known from Mexico, but up to 2006 a total of 28 confirmed cases were recorded in Texas. Of these, 24 cases were associated to SNV and 3 to BAYV. Infection with SNV had a high mortality rate of 50% (12/24), but all of the three patients infected with BAYV survived (95). The geographic distribution of HPS cases in Texas shows the two groups with most cases (64%) are in the West and Panhandle areas, with the rest of the cases found along the Gulf of Mexico Coast area. This disjoint case distribution is due to rodent reservoir distributions. Cases along the Gulf Coast are associated to the BAYV, which is carried by the rice rat (*Oryzomys palustris*), whereas the cases in Western Texas have been related to SNV presence in the deer mouse (*P. maniculatus*) (95). *O. palustris* is present in Northeastern Mexico, but no seropositive individuals have been reported. Its role as a BAYV reservoir in Mexico is likely minor as the range of this rodent is limited to the extreme Northern corner of Tamaulipas (90). However, two rodent species (*P. maniculatus*, and the white-footed mouse, *Peromyscus leucopus*) pose a more serious risk for HPS along the transboundary States. Both species have wide ranges across Texas and Northeastern Mexico; within this region, SNV seroprevalent individuals have been recorded for both species (88, 94, 95). Moreover, based on the list of Hantaviruses identified in the Southwestern US, it is suggested that four Hantaviruses likely circulate in Northern Mexico: SNV, ELMCV, MULV, and Limestone Canyon virus (LSCV). These viral strains are hosted by rodent species of the *Peromyscus*, *Reithrodontomys*, and *Sigmodon* genera (88), which are ubiquitous throughout the transboundary region. This hypothesis needs to be confirmed with further work to ascertain the public health risk for human populations on both sides of the border. To achieve this goal, it is necessary to determine the geographic distribution patterns of *Hantavirus* sero-prevalence in rodent reservoir species and understand the mechanistic processes that determine these patterns.

Beyond knowing the specific situation in the Eastern transboundary region between Mexico and the US, further work needs to extend outside of this zone, as it might influence this region. Mexico has a very diverse mammalian fauna (~525 species) with rodents comprising almost half (235 spp., 44.8%) of the species (90). It is possible that many more species harbor Hantaviruses than those currently recognized (88). Specifically, the transboundary region could serve as a connection between Hantaviruses of Neartic origin with others from tropical regions. The State of Tamaulipas has a mammalian fauna that represents a mix of Neartic and Neotropical taxa (90) and the possibility exists that these rodent species of Neotropical affinities could harbor Hantaviruses found in tropical areas such as the Catacama virus (CATV) present in *O. couesi* from Honduras (83, 87). Moreover, evidence of the presence of *Hantavirus* seropositive rodents in regions of Southern Mexico and crucial human migration crossing pathways are elements to be considered in this complex equation (88). For instance, in the State of Chiapas’ coastal and central valleys, there are clear indications for the presence of *Hantavirus* in wild life. After infection, the resultant disease can take up to 2 weeks to develop in the human host, allowing the disease to move with relatively low detection. Thus, the potential risk of these Neotropical Hantaviruses existing in the transboundary region needs to be evaluated.

### Bacterial infectious diseases

#### Tick-borne bacterial infections

Globally, ticks serve as vectors for a number of zoonotic bacterial pathogens, such as the spirochete *Borrelia burgdorferi*, the causative agent of LD, as well as the intracellular pathogens *Rickettsia rickettsii*, *Ehrlichia chaffeensis*, *E. ewiingii*, and *Anaplasma phagocytophilum* (Table 1), also known as tick-borne rickettsial diseases (TBRD) (96). These pathogens are maintained in natural cycles involving wild mammals and several species of hard ticks in the family Ixodidae. Foci of LD exist in the US, Europe, and Asia, and it is considered an emerging infection in those parts of the world (97–101). In the US LD is the most prevalent arthropod-borne infection with over 30,000 cases reported to the Centers for Disease Control and Prevention (CDC) in recent years. The increase in LD cases during the last few years has prompted its classification as an emerging infectious disease. Similar to other arthropod-borne diseases, LD is a complex system subject to shifts in ecological processes that influence vector biology and the epidemiology of *B. burgdorferi* infection in reservoir hosts and humans.

Hard tick species in the genus *Ixodes* are recognized generally as common vectors of *B. burgdorferi*. *I. scapularis* and *I. pacificus* are known competent vectors in the US, while *I. persulcatus* and *I. ricinus* are the documented vectors in Eurasia (99, 102–105). The pathogen is maintained in the environment by different vertebrate hosts with varying degrees of competence. The main reservoir, the white-footed mouse, *Peromyscus leucopus*, is found in the forests of Eastern North America (106, 107). There is an extensive bibliography on the molecular diversity and adaptation of *B. burgdorferi* to its natural environment, as well as on the impact of species diversity in a particular area on reducing LD risk (108–118). In addition, some of these studies have also considered the effect of climate change on the geographic distribution of *I. scapularis* in addition to its phenology in the US and Canada (119–121). Although LD in humans is more prevalent in Northeastern US, the lack of detailed studies in Southern US has prevented comparisons, and evaluations of ecological factors responsible for promoting the differential incidence of LD between these regions. Moreover, in some parts of the world the ecology and epidemiology of LD remain to be fully understood. Thus, LD is considered a transboundary zoonotic disease in that it can reach epidemic proportions in regions of the globe regardless of country borders (122). This is coupled with the fact that there is unequally distributed knowledge about the ecology of this disease among the regions in which it occurs.

Human risk of infection with *B. burgdorferi* across the continental US has been predicted using the density of *I. scapularis* infected nymphs (DIN) (123, 124). Under this scenario, Southern US States were considered as a low risk region given the non-appearance of host-seeking *I. scapularis* nymphs at sampled sites (123, 124). In striking contrast to the conclusion of this suggested null risk of acquiring LD in Southern States, a steady number of LD cases have been reported in these low risk areas every year (125, 126). Some of the caveats of these most recent studies include the lack of accounting for both human movement (some cases can be acquired in a region different from the one where they are reported) and differences in tick phenology between geographic areas (124). These limitations might explain why the models utilized cannot explain the variation in distribution of the disease observed in low incidence areas. Drivers for the variation in distribution of disease cases observed in low incidence areas remain to be identified.

In Mexico, a national serosurvey of human serum samples reported a *B. burgdorferi* sero-prevalence of 1.1% (127). The Mexican States of Tamaulipas, Nuevo León and Coahuila in the Texas–Mexico border region presented the highest sero-prevalence (6.4%) for the country (128). Also, *Ixodes* ticks infected with *B. burgdorferi* sensu stricto occur in the same States (129), and recently the infection has been documented in white-tailed deer (130). Distribution models of potential tick vectors in Mexico point to a wide distribution range that overlaps not only Northeastern Mexican States along the border with the US, but also extend to central Mexico (131, 132). These studies, together with confirmed clinical cases of LD acquired in parks near Mexico City (133, 134), demonstrate the existence of a zoonotic cycle responsible for LD in Mexico.

TBRD are a group of zoonoses clinically similar, yet epidemiologically and etiologically distinct. In the US, these diseases include: (1) Rocky Mountain spotted fever (RMSF), (2) human monocytic ehrlichiosis (HME), (3) human granulocytic anaplasmosis (HGA) (135), (4) *Ehrlichia ewingii* infection, and (5) other emerging TBRD (Table 1). TBRD are common occurrences in the medical and veterinary clinical setting, and are gaining more attention from physicians and veterinarians since TBRD continue to cause severe illness and death in otherwise healthy individuals (136, 137). The epidemiology of these diseases reflects the geographic distribution and seasonal activities of vectors and reservoirs and human behavior that places persons at risk for infection through tick bite (13, 129, 137, 138). Environmental changes may alter the distribution of wild animals and arthropod vectors, which could extend their range to areas close to human populations where these pathogens could be transmitted (13). But demographic and sociologic factors also play a critical role in determining disease incidence.

Several ticks species are vectors of different rickettsiae causing TBRD. *R. rickettsii*, the causative agent of RMSF, is transmitted most frequently by the American dog tick (*Dermacentor variabilis*) in the Eastern, Central, and Pacific coastal US and the Rocky Mountains, while the wood tick (*D. andersoni*) transmits this pathogen in the Western US. The brown dog tick (*Rhipicephalus sanguineus*), a vector of RMSF in Mexico (129, 137, 139), was implicated in 2005 as vector of this disease in a confined geographic area in Arizona (140). *Rhipicephalus* ticks from Mexicali, Mexico have been recently genetically characterized, and found to be different from those isolated in the US (141). The cayenne tick (*Amblyomma cajennense)* is a common vector for RMSF in Central and South America (129, 132, 142), and its range extends into the US through Texas. *Ehrlichia chaffeensis* and *Ehrlichia ewingii* are transmitted to humans by the lone star tick (*Amblyomma americanum*). *E. ewingii* infections in dogs or ticks have been described in Missouri, Oklahoma, Tennessee, Arkansas, Texas, Florida, Georgia, Mississippi, North Carolina, and Virginia (143, 144). *A. phagocytophilum* is transmitted by the blacklegged tick (*I. scapularis*) and is distributed in New England, North Central, and recently, Southeast United States, in addition to the Northeast of Mexico (13, 129). The western blacklegged tick (*I. pacificus*) is the principal vector in Northern California. In the US, the estimated average annual incidence of RMSF was 2.2 cases per million people. In Mexico, the incidence from 1975 to 1987 was 12.59 cases per 100,000 people in North and Northwest States. From 2009 to 2011, there were 2616 reported cases with an incidence of 0.8 cases per 100,000 people. In 2012, there was an increase to 2875 cases in the States of Baja California and Coahuila in Northern Mexico. Due to the consistent increase and presence of this disease, Mexico started to officially report RMSF and other Rickettsial human cases in 2014 (145).

Ehrlichiosis was first recognized as a disease in the late 1980s, but did not become a reportable disease until 1999 in the US. The number of ehrlichiosis cases due to *E. chaffeensis* that have been reported to CDC has increased steadily since the disease became reportable, from 200 cases in 2000 to 961 cases in 2008 (138). The incidence increased from less than 1 to 2.5 cases per million people in 2000–2010. Both *E. chaffeensis* and *E. ewingii* are causes of human illness in the US, although the majority of reported cases identify *E. chaffeensis* as the causative agent of HME. HGA is more frequently reported than HME with an annual incidence of 1.6 cases per million during 2001–2002. In Mexico, the first Ehrlichiosis case was reported in 1999 (146). It is important to understand the involvement of dogs in the potential enzootic cycle for *Ehrlichia* infection acquired by humans in close contact with domestic dogs. In this sense, our research team found that human contact with *Ehrlichia* infected dogs have 14.9 times higher risk to become infected, and dogs infested with *Ehrlichia* infected ticks have 8.2 higher risk of being infected (128).

The understanding of vector-borne disease ecology has improved in recent years due to advancements in molecular biology, geographic information systems (GIS), and species distribution models (SDM) (147, 148). A recent study evaluated the presence of *I. scapularis* ticks in Texas and Northern Mexico, and forecasted the distribution of this tick species considering different climate change scenarios (13). It was observed that a geographic region could provide suitable environment where the competent vector for transmission of LD and other zoonotic pathogens would survive. The model presented in this study showed East Texas to include suitable habitat where established populations can exist (13), which agrees with findings from other studies (149). This model also showed expansion towards Central and to South Texas through a corridor along the Gulf Coast and Northern Mexico, forming a geographic continuum of habitat suitable for *I. scapularis* populations in the border region. Although no specific distribution model exists for *I. scapularis* in Mexico, a distribution model for the genus *Ixodes* generated with similar methodologies predicts a wide distribution covering Northeastern Mexico (132).

Variation in questing behaviors may significantly impact the type of hosts ticks encounter and may lead to differential host use within a particular study area (150). Therefore, further studies testing different sampling procedures, including different time of the day and season, would be needed to determine the phenology of *I. scapularis* in Southern US and Mexico and the questing behavior of the different developmental stages. These studies will be critical to determine how questing behavior will affect the risk for LD in humans and companion animals in the transboundary region.

#### Leptospirosis

Leptospirosis is a zoonotic infectious disease of worldwide distribution that is endemic in tropical and temperate climates, with higher prevalence in tropical countries (151–153). Leptospirosis can be caused by *Leptospira interrogans*, which in cludes 200 serovars affecting both domestic and wild mammals, and humans (153, 154). The reservoirs for these pathogens are wild or domestic animals such as rodents, cattle, or dogs (155). In urban areas, rodents (mostly rats) are the main carriers of the disease (156), whereas the dog is considered a dead-end host (157, 158). However, due to their close contact, dogs pose a risk of infection for human beings (151, 159). It has been suggested that ticks are potential vectors of *Leptospira* spp. (160).

In the forest region of Indiana, a study was conducted with 34 raccoons (*Procyon lotor*) to determine the presence of *Leptospira*. In this study, cell culture techniques, microscopic agglutination test (MAT), and PCR were used. The results indicated the presence of *L. interrogans*, *L. kirschneri*, and *L. borgpetersen* in raccoons from this region. For *L. interrogans*, the serovars most frequently detected were Bratislava (38.2%) and Grippotyphosa (32.4%). This finding indicated that *L. interrogans* is circulating in the raccoon population, which is acting as a reservoir for the pathogen. The racoon is an abundant species in the south Texas – northeast Mexico region.

Cervids may be involved in the epidemiology of leptospirosis. Diversified livestock production comprises activities aimed to breed in sustainable manner wild animals, including native and exotic species of deer. Ranches in northeast Mexico have been managed to be units for the conservation, management, and sustainable use of wildlife (UMA), which are dedicated to the diversification of livestock. A cross-sectional epidemiological study on leptospirosis was conducted with cervids at an UMA in Tamaulipas, Mexico (161). Of the 37 animals sampled, eighteen individuals were Axis deer (*Cervus axis*) and 19 Fallow deer (*Cervus dama*). Seropositivity for *Leptospira* spp. in all the cervids sampled was 13.5%. Twenty-one percent of the Axis, and 5.5% of the Fallow deer were seropositive. Positive deer were reactive to serovars Bratislava and Muenchen, which confirms the presence of this pathogen in deer in Northern Mexico (161). Similar seroepidemiological findings for leptospirosis in cervids have been reported in the US and Spain (162–165).

It is important to understand how global change may alter the pathogenic landscape of leptospirosis because this zoonosis is considered a major bacterial NTD in Texas and Mexico (11). For example, the water buffalo (*Bubalus bubalis*) is originally from Asia and was introduced in Mexico as an alternative livestock species during the 1990s (166). Water buffaloes produce milk, meat, and are used as working animals. Although subclinical *Leptospira* infection has been documented in other parts of the world and is considered a health risk to humans, information is lacking on the prevalence of leptospirosis in water buffalo herd in Mexico. An epidemiological cross-sectional study was conducted with a sample size of 368 blood specimens in the Mexican state of Veracruz to fill this knowledge gap. The overall sero-prevalence for Leptospirosis was 53.5% (167). The most common serovars detected were Muenchen (44.3%), Pyrogenes (11.4%), Icterohaemorragiae (11.1%), and Hardjo (8.1%). In this study, the interaction between water buffaloes and dogs was identified to be a risk factor (167). This was the first study identifying seropositive buffaloes to *L. interrogans* and risk factors associated with Leptospirosis in Mexico (167).

Zoonoses common to pets threaten the health of humans, particularly children. A study conducted to determine the frequency of canine Leptospirosis in dogs from two shelters in the city of Veracruz, Mexico showed that 8.6% (8/92) were seropositive. The most frequent serovar was Canicola (168). Similar results were observed in Yucatan, Mexico where the serovars present were Canicola and Icterohaemorrhagiae (169). Even though leptospirosis occurs in the transboundary region, more research is required to understand its epidemiology and mitigate the burden of this neglected zoonosis on human and domestic animal populations (11).

#### Bartonellosis

*Bartonella* species are fastidious gram-negative, facultative intracellular bacteria that cause host restricted hemotropic infections in mammals. Normally, they infect erythrocytes, macrophages, and endothelial cells. In addition, a number of *Bartonella* spp. are transmitted by blood sucking arthropods such as sand flies, biting flies, lice, and fleas. More recently, ticks and mites have been suggested as potential vectors for zoonotic *Bartonella* spp. (170–175). In addition, *Bartonella* spp. affect a number of mammals, including humans, dogs, horses, cattle, cats, and even marine animals (176, 177).

The number of *Bartonella* spp. identified infecting a wide range of mammalian species has significantly increased. Currently, a total of 13 species or subspecies can cause disease in humans, and most of them are zoonotic (173, 176, 178). *Bartonella henselae*, the causative agent of cat-scratch disease (CSD) is the most recognized in the medical community together with *B. quintana*, the causative agent of Trench Fever, and *B. bacilliformes*, the causative agent of bacillary angiomatosis, Oroya fever and veruga peruana (176, 177). Moreover, most of the *Bartonella* species causing disease in humans and companion animals have a worldwide distribution, and are associated with poverty and overpopulation, as these bacteria thrive in crowded and unhygienic environments (172, 179–181). This aspect of *B. quintana* and *B. elizabethae* infections has been extensively reviewed elsewhere (180–184).

Cats have been classified as reservoirs for *B. henselae*, the causative agent of CSD, which is distributed worldwide. The prevalence of infection in felines is higher in warmer countries that it is in cold countries (178). In addition, cats tend to be bacteremic for months and in some instances for over a year (178). The cat flea (185), *Ctenocephalides felis*, is responsible for the transmission of the bacterial pathogen between cats. Studies by Finkelstein and collaborators (180) showed that *B. henselae* can remain viable in flea feces for over 72 h. Therefore, transmission to humans can occur via inoculation of *B. henselae* from infected flea feces into the skin via open wounds, such a scratch lesion. The CDC documents over 20,000 CSD cases annually. Human cases have been reported in Texas (186–189), Mexico, and other Latin American countries (190, 191). Domestic dogs, together with wild canids, have been suggested as potential reservoirs for zoonotic *Bartonella* species, such as *B. vinsonii* subsp. *berkhoffii*, *B. henselae*, *B. clarridgeiae*, *B. wahoensis*, *B. rochalimae*, *B. quintana*, and *B. elizabethae*. In some cases, domestic dogs display a broad range of clinical signs (174, 176, 178, 192, 193) similar to those observed in humans. Consequently, domestic dogs might be considered sentinels for *Bartonella* infections (178) as they are for other vector-borne infectious diseases (194–196).

The number of annual cases due to Bartonella infection in the US–Mexico transboundary region remains unknown even though more epidemiological information is available relative to bacterial NTD like leptospirosis (197). *Bartonella* infections are not reportable in humans or animals in the US and Mexico. This situation makes it difficult to understand the epidemiology and pathogenic landscape for Bartonellosis in a geographic region where about 17.6% of the population lives in poverty^2^.

### Protozoan infectious diseases

#### Human babesiosis

Human Babesiosis (HB) is caused by several species of apicomplexan tick-borne protozoa of the genus *Babesia* and is a zoonotic emerging disease globally although it is endemic in the US (198–200). Surveillance for Babesiosis started in 2011 in 18 States. That year, there were 1,124 confirmed and probable human cases across the US (126). Most cases of naturally transmitted HB in the Northeastern and Midwestern US are caused by *B*. *microti* (201). Infection in other regions of the US and the world has been documented with *Babesia* species that remain to be fully characterized (202–204). In rural Mexico, human infection with *Babesia* spp. was documented during the 70s (205), where 37% of the tested individuals were seropositive but asymptomatic, and all volunteers recalled having tick bites.

HB is a complex zoonotic disease system whose relevance as an emerging public health concern is considered to be the result of aspects related to global change (206, 207). Most zoonotic *Babesia* species are maintained in wildlife reservoirs, but gaps exist in our knowledge of several epidemiological aspects of HB in various parts of the world (208–210). The vector(s) and reservoir hosts of *Babesia* species affecting humans in Mexico and other Latin American countries remain unknown (211). Accurate diagnosis of HB in sub-tropical and tropical parts of Latin America can be complicated because of malaria-like symptoms in the case of affected patients (212), asymptomatic infection in others, and the possibility that tick bite may have resulted in co-infection with other tick-borne pathogens (213, 214) coupled with the use of serodiagnostic tools that cross-react with other *Babesia* and *Plasmodium* hemoparasites (215, 216). Thus, it is important to understand how environmental change (217), including the knowledge of vectors and reservoir hosts, could influence the patterns of zoonotic *Babesia* transmission at the regional level to evaluate the risk for the emergence of Babesiosis among humans in the South Texas–Northeast Mexico transboundary region.

Although its known geographic range appears to be expanding, it is possible that HB remains under-reported in the South Texas – Northeast Mexico transboundary region (218–220). Increasing evidence suggests that reservoir species and vectors are present in the Eastern sector of the border between the US and Mexico, and our knowledge of wildlife and domestic animals harboring *Babesia* species with the potential to be pathogenic to humans is expanding (204, 221, 222). The infection of cotton rats (*Sigmodon hispidus*) and raccoons (*Procyon lotor*) with *B*. *microti* presents a risk for HB in Northeast Florida (223). Both mammals are abundant in Texas and Northeast Mexico (129, 142, 224, 225). *I*. *scapularis* is the known vector in the Northeastern US and its presence has been documented in the US–Mexico transboundary region (13), but Clark et al. (223) suggested that additional *Ixodes* species might be transmitting *B*. *microti* in the Southeastern US, like the raccoon tick (*I*. *texanus*) and other *Ixodes* ticks known to feed on the cotton rat. *I*. *texanus* is distributed in Texas and Nuevo León (142, 226, 227). Of the ticks species present in the US and Mexico, which are known to infest the cotton rat (13, 228–230), *D*. *variabilis* and *I*. *scapularis* have been shown to harbor *Babesia* spp. and bite humans in Southern latitudes (231, 232). Suspected vectors of a *Babesia* detected in the woodrat (*Neotoma micropus*) with a partial 18S rRNA sequence related to *B*. *conradae* included *A*. *inornatum* and *D*. *variabilis*, which are ticks reported to parasitize humans in Texas (233, 234). *A*. *inornatum* is found in the Mexican border States of Coahuila, Nuevo León, and Tamaulipas (142, 235). Blood donors from Texas were seropositive for *B*. *duncani*, but the vector and reservoir host species remain to be determined (236). Notably, four adult *A*. *americanum* and one adult *D*. *variabilis* PCR positive for *Babesia* spp. were removed from humans in Texas (231). One of the *A*. *americanum* was infected with a large *Babesia* molecularly resembling a large *Babesia* detected in an immunocompromised dog residing in Texas (237). Nevertheless, the zoonotic potential of the other *Babesia* infecting *A*. *americanum* remains to be determined.

Global change is altering the distribution of ticks and tick-borne diseases globally, and the South Texas–Northeast Mexico transboundary region is not immune to this process (13, 198, 238). Environmental drivers for the emergence of HB as a public health concern in other parts of the world seem to be at play in the Texas–Mexico border (219, 231, 232). Assessing the incidence of HB accurately requires knowledge of the rates for human-tick contact and infection in the vector population. However, HB is considered to be under-reported, even in states with a surveillance program and areas where the disease is known to be endemic (201). Diagnosis can be complicated because of co-infection with *B*. *burgdorferi* and *Plasmodium* spp. (239, 240). It remains to be determined if co-infection with *B*. *burgdorferi* and *Babesia* spp. affecting humans occurs in populations of *I*. *scapularis* inhabiting ecosystems spanning the US–Mexico border. However, the presence of potential vectors and reservoirs indicates that studies are required to determine if zoonotic Babesiosis is an unrecognized cause of illness among humans in that transboundary region. Tick-based surveillance has been proposed as an alternative approach to assess infection risk because it provides a more sensitive method for identifying areas where Babesiosis could be emerging, and could be used to estimate zoonotic prevalence in established areas (218). The adaptation of this strategy, with an international perspective and in the context of the One Health concept is suggested, as it was done recently for LD, to establish an early warning system for the emergence of HB in the Texas–Mexico border region (13, 241).

#### Leishmaniasis

Leishmaniasis is a vector-borne disease caused by *Leishmania* species of the family Kinetoplastidae, which is transmitted by sand flies of the genus *Lutzomyia* in the Americas and *Phlebotomus* in other regions of endemicity (242, 243). There are 98 countries where *Leishmania* is endemic, with the majority of cases occurring in developing nations (244). The distribution of competent vector species and leishmaniasis has expanded over the last decade as areas with suitable habitat for sand flies continue to increase due in part to shifts in climate (14). Of the 1.6 million new cases per year estimated to occur worldwide, approximately 600,000 are recorded (245). Moreover, leishmaniasis is estimated to affect about 12 million people in four continents (Africa, Americas, Asia, and Europe) (245, 246).

The leishmaniases have been divided in two main syndromes: Old World, and New World leishmaniasis (247). Old World leishmaniasis includes two clinical presentations: cutaneous leishmaniasis, which is confined to skin, and visceral leishmaniasis, which involves the bloodstream and inner organs. New World leishmaniasis’ clinical presentation can manifest in a cutaneous form, or as a mucocutaneous syndrome, which involves mucous membranes in addition to the skin (245, 246). Presently, new terms are used to describe the clinical forms of leishmaniasis. The term mucosal leishmaniasis indicates the involvement of mucosal tissues such as mucous membranes of the upper respiratory tract and oral cavity, i.e. mucocutaneous leishmaniasis (246). Together with the cutaneous and diffuse cutaneous forms the mucocutaneous syndrome is one of the typical presentations of leishmaniasis in South America (248).

Twenty-one *Leishmania* species have been identified as human pathogens. They are systematically classified in four complexes. In the New World leishmaniasis is caused by species belonging to the subgenus *Leishmania* [such as *Leishmania (Leishmania) mexicana*, *L*. *(Leishmania) amazonensis*] and the subgenus *Viannia* [*L*. *(Viannia) braziliensis*, *L*. *(Viannia) panamensis*, and *L*. *(Viannia) guyanensis*] (246, 249). In Mexico and US, cutaneous leishmaniasis is caused by a number of *Leishmania* spp. with widespread distributions and a variety of location-specific reservoir species (242). Numerous species causing cutaneous leishmaniasis have been identified in multiple mammalian species. *L. mexicana* is found from Central America to the Yucatan peninsula in Mexico, and cases have been reported in Texas (250). In the Old World, *Leishmania major* is a predominant cause of cutaneous Leishmaniasis.

In Mexico, the first clinically documented records of cutaneous leishmaniasis were from forested parts of the Yucatan Peninsula (251, 252). Until 1989, only eight cases of visceral leishmaniasis were reported; all of them were in the Balsas River basin, which includes the States of Guerrero, Puebla, Morelos, and Oaxaca (253). In Chiapas State, the first case was documented in Tuxtla Gutiérrez in 1990. An increase in cases in several municipalities was observed in subsequent years. From 1990 to 2006, 89 cases of American visceral leishmaniasis were reported in Chiapas State (254). In the US, human cases (*n* = 30) of non-travel-related (or autochthonous) disease have been reported since 1903 in the epidemic focus located in South-Central Texas. In 2008, nine cases of non-travel-related cutaneous leishmaniasis in Northern Texas, specifically in suburbs and smaller towns near the Dallas-Ft. Worth metro area, were reported (255). Subsequently, four cases of autochthonous cutaneous Leishmaniasis were described in Northeastern Texas and Southeastern Oklahoma (256).

Several *Leishmania* species are transmitted zoonotically, and in the case of *L. infantum*, dogs are the main reservoir. In many settings, dogs may serve as a link between sylvatic and domestic cycles of visceral leishmaniasis. Dogs can cross forest-edge boundaries, thereby potentially bringing parasites to or from sylvatic systems and to and from other potential mammalian hosts (such as foxes, rodents, and opossums) (242). In Yucatan, Mexico, the prevalence of *L. mexicana*, *L. infantum*, and *L. braziliensis* in dog sera (*n* = 218) was 30.2, 11.9, and 8.2%, respectively (257). Antibody based prevalence of 10.5% for *L. mexicana* and 11.57% for *L. braziliensis* has also been reported in cats (258). Vertical transmission of leshmaniasis has been characterized for dogs and people, causing an increased risk for infants born to parasitemic mothers (259). There have also been a number of non-travel-associated reports of cutaneous leishmaniasis in companion animals in Texas (250). Many of these cases of zoonotic cutaneous leishmaniasis were in cats, which may be associated with an outdoor life-style (250). Until recently, visceral leishmaniasis was thought to be primarily an imported disease in North America. Infected dogs had usually been imported from regions in Southern Europe or South America where *L. infantum* and *L. chagasi* were enzootic (260, 261). Additional risk factors for humans are related to their immunologic status and their ability to clear infection or maintain an asymptomatic state. These factors include concurrent infection with HIV, co-infections with helminthic parasites, drug abuse, and other immunosuppressive conditions (262).

A serosurvey conducted in the US looked at over 12,000 foxhounds and other canids, as well as 185 people in 35 States, to determine geographic extent, prevalence, host range, and modes of transmission. This study showed that foxhounds infected with *Leishmania* spp. were present in 18 States. However, no evidence of infection was found in humans (263). While companion animal infection and transmission occur, the predominant sylvatic reservoir in Texas is the Southern Plains woodrat, *Neotoma micropus* (264). Given the presence of sand fly vectors throughout the Southern US, it is possible that disease rates associated with *L. mexicana* infection will increase in the US due to climate change (14). South American species causing cutaneous leishmaniasis, including *L. amazonensis*, *L. braziliensis*, *L. guyanensis*, and *L. panamensis*, have sylvatic reservoirs (242). Thus, human risk factors for zoonotic cutaneous leishmaniasis are dependent upon exposure to vector species and the presence of reservoir species. Urbanization and wilderness encroachment have resulted in increased interactions between humans, reservoir, and vector species and the establishment of peri-urban domestic life cycles rather than sylvatic ones (242).

In Mexico, *Lutzomyia olmeca olmeca*, *Lu. cruciata, Lu. shannoni, Lu. panamensis*, and *Lu. ylephiletor* have been incriminated as vectors of *Leishmania* spp. (14, 265). In Northern Mexico and US, sand fly species suspected of being involved in *Leishmania* transmission to humans are *Lu. diabolica* and *Lu. anthophora* (266, 267). *Lu. diabiolica* is suspected of being a vector of *L. mexicana* and has been infected experimentally with *L. infantum*. In addition, *Lu. anthophora* was able to transmit *L. mexicana* experimentally to mice (268, 269). *Lu. shannoni* is a possible vector of *L. infantum*, and is present in the Midwestern, Southern, and Southeastern US (263). The sand fly vectors of *L. infantum* causing visceral leishmaniasis in Mexico include *Lu. longipalpis* and *Lu. evansi* (270). Visceral leishmaniasis cases have been reported in the States of Chiapas, Guerrero, Puebal, Oaxaca, morelos, and Veracruz where dogs are considered to be disease reservoirs (270).

The pathogenic landscape for leishmaniasis in the transboundary region remains to be fully understood (271). In Mexico and the US, the risk for leishmaniasis has been associated with forest habitat like pluvial rainforest and agricultural fields close to the forest where reservoir mammals share habitat with humans. However, the incidence of leishmaniasis is increasing in domestic habitats as a direct consequence of the spreading of sand fly vectors to urbanized areas, especially the outskirts of cities (272). Moo-Llanes and collaborators (271) studied the current and future niche of North and Central American sand flies and concluded that continued landscape modification and future climate change will provide an increased opportunity for the geographic expansion of sand flies and increased risk for human exposure to *Leishmania* infection. The One Health paradigm could also be applied to enhance our ability to recognize *Leishmania* spp. in humans, domestic and wildlife reservoirs, and sand fly vectors in the US-Mexico transboundary region (273).

#### Chagas

Chagas is a zoonosis caused by *Trypanosoma cruzi*, a protozoan parasite that is present in a variety of mammalian reservoirs. This disease is one of the most prevalent parasitic diseases in the world (274) and kills around 45,000 people annually (275). The parasite is transmitted by species of insect vectors, commonly known as kissing bugs, belonging to the sub-family Triatominae in the family Reduviidae (276, 277). During the blood meal, the Triatomine kissing bug defecates and sheds the parasite in the feces. The parasite then enters its host through the bite wound or through contact with mucous membranes. The parasite can also be transmitted through blood transfusion, organ transplants, ingestion of infected food, or congenital transfusion (277). However, 85–96% of *T. cruzi* transmission to humans occurs via contact with infected feces from Triatomine insects (278). The acute phase of the disease is rarely recognized since cases are typically subclinical and asymptomatic. Chagas disease can then enter the chronic phase, in which 30% of cases will develop a fatal cardiomyopathy around 10–30 years post-infection (274, 279). Anti-parasitic treatment is mostly effective during the acute phase, and in infants and individuals up to 15 years old, although the currently accepted therapeutic options have limited efficacy and can have disabling side effects. Current research includes efforts to develop a vaccine for Chagas (11).

Chagas is endemic in the Southern US and Latin America, where it affects more than 10 million people (280) and it is spreading rapidly to non-endemic areas (276). It is considered a NTD in Texas (11), and Chagas could have been present in hunting-gatherer native Americans as far back as 1200 years ago, as described in a case of megacolon found in a mummified ancient resident of what is now known as Texas (281, 282). Studies conducted in US blood donors have demonstrated that *T. cruzi* seropositive donors have persistent infection with demonstrable parasitemia long after acquisition of infection (283). In Texas, for example, there are an estimated 267,000 people infected (284, 285). This is only an estimate based on small sero-prevalence studies and risk modeling. The exact risk for infection and the number of Chagas cases in Texas is unknown (277). In January 2013, Chagas became a reportable condition in Texas, which is a critical step toward documenting cases and understanding the epidemiology of this critical NTD.

There are several Chagas endemic areas in Mexico, including the States of Yucatán, Chiapas, Guerrero, Oaxaca, Jalisco, Veracruz, Puebla, Hidalgo, and Morelos where the disease occurs mainly in rural areas (286). The highest prevalence was observed in the Northeastern region of the country, which corresponds to the central area of a tropical region comprising the States of Hidalgo, San Luis Potosí, Veracruz, and the US neighbor State of Tamaulipas (287, 288). Recent cases of Chagas have been reported in Coahuila (289, 290) where *T. cruzi* infection has been found also in blood donors (290). There have been new records of *T. gerstaeckeri* and *T. rubida* in Nuevo Leon and Coahuila (291). As in the US, *T. cruzi* is increasingly transmitted through blood transfusions partly due to migration from rural areas toward Mexico City (292). More than 180 domestic, synanthropic, and wild species of mammals, especially rodents and marsupials, are likely to be infected with *T. cruzi* and to be involved in the disease transmission cycle (293). In the Yucatan peninsula, the anti-*T. cruzi* antibody prevalence in dogs and cats were determined to be 14.76 and 4.21%, respectively (258, 294). In addition, an active canine *T. cruzi* transmission cycle with severe symptoms affecting a broad range of dog breeds and age groups was observed in several counties in Texas (295). Further, in regions of Central Mexico, studies have demonstrated that canine sero-prevalence is directly correlated to human sero-prevalence, demonstrating the importance of this host as a sentinel species (296). Serosurveillance in shelter dogs was found useful as a public health tool to assess the risk for *T. cruzi* infection (297).

Currently, 40 species of triatomine insects are known to be naturally infected with *T. cruzi* in North America. Twenty-eight species are found exclusively in Mexico, and eight are shared with the US (298). Considering the vectorial transmission capacity and widespread distribution in Mexico, important species include *Triatoma barberi*, *T. dimidiata*, *T. phyllosoma*, *T. longipennis*, *T. mazzottii*, *T. pallidipennis*, *T. picturata*, *T. mexicana*, and *T. gerstaeckeri* (299, 300). *T. gerstaeckeri* and *T. sanguisuga* are the most common triatomine species in the Southern US and might be involved in *T. cruzi* transmission. Different reports have revealed information about the major vectors in endemic areas of Mexico (287). In the State of Veracruz, the main recognized vectors are *T. dimidiata* and *R. prolixus*, and three vector species, *P. rufotuberculatus*, *R prolixus*, and *T. dimidiata*, have been identified in the State of Chiapas. Two important vectors in the Southern region of the State of Mexico are *T. pallidipennis* (97.4%) and *T. dimidiata* (2.6%), and 28.0% of the triatomines in that region were infected with *T. cruzi* (299). Studies conducted in rural communities of Yucatan, Mexico found that 21.9% of *T. dimidiata* (23.9% of adults and 13% of nymphs) were infected with *T. cruzi* (301).

An accepted strategy to control and prevent Chagas disease is through the management of triatomine insect infestations in domestic areas based on government and population surveillance programs (302). These programs have been successful in South America, including Mexico. However, a surveillance program aimed at controlling triatomine vectors and preventing Chagas disease has not been attempted in the US, perhaps due to the lack of data on human cases and vector-parasite distribution. Triatomine insects can be found in domestic and sylvatic life cycles, which could make efforts to control insect infestations difficult in domestic habitats due to the continuity of insect populations (303).

Recent models of risk assessment have identified Hidalgo and Cameron counties as the areas of highest risk for human infection of Chagas disease in Texas (277). Hidalgo County has suitable climatic conditions for the vector (304). This risk factor could be compounded by a high poverty rate among the border population and substandard housing. In Hidalgo County alone, almost 130,000 people live in unincorporated residential areas known as *colonias*. Much of this population is characterized by low incomes (<$10,000 per year) and poorly constructed residences with substandard sanitation and drainage systems, which are landscape characteristics that provide suitable habitat for Triatomine insects (11, 277). Hidalgo and Cameron County form part of the Lower Rio Grande Valley (LRGV), one of the main points of entry for immigrants from Latin America, mainly Mexico. Since Chagas disease is endemic to tropical areas, it is possible that migrants can carry the disease into the US via the LRGV (11). Because Chagas became a reportable disease in Texas in 2013, it can be argued that sufficient epidemiologic data are lacking to implement a scientifically sound disease control and prevention program yet. To be effective, a management program to control and prevent Chagas disease in south Texas will probably need to be combined with government and population-based surveillance of insect infestation. Important challenges to overcome include a lack of knowledge among the local population about Chagas disease and how to identify insect vectors.

Climate change could drive enhanced transmission of *T. cruzi* (12). A potential Northern shift from current range due to climate change could occur with two of the most common triatomine *T. cruzi* vectors in the Southern US (*T. gerstaeckeri* and *T. sanguisuga*). Furthermore, an increase in temperature may have influenced the behavior of triatomine species (305, 306). When temperature exceeds 30°C and humidity is low, the insects increase their feeding rate to avoid dehydration. In addition, in domestic life cycles, when indoor temperatures increase, the insects may develop shorter life cycles and reach higher population densities (305). High temperatures could also speed up the development of *T. cruzi* in vectors (307).

*Trypanosoma cruzi* has three infective forms capable of infecting its host and currently six DTUs (discrete typing units) are recognized in the taxon. These DTUs correlate with mammalian hosts specific interactions in distinct time-space scales. We know relatively little about confirmed mammalian *T. cruzi* hosts in the US. More studies are needed to produce a comprehensive list of confirmed *T. cruzi* hosts as well as time-space scales for the operative interactions of hosts, vectors, and parasites. To better understand the epidemiology of Chagas disease in the Texas–Mexico transboundary region, ongoing research could focus on detecting *T. cruzi* infection status of vectors, potential role of the different reservoirs and hosts in the parasite cycles, and DTUs identification (12).

## Concluding Remarks

The threat of zoonotic diseases to human and animal populations in the Mexico–US border along the Rio Grande is documented here. Attention is called to a gap in understanding of the pathogenic landscape for zoonotic vector-borne diseases in this transboundary region. Among other things, research on ecosystem processes, land use, and human behaviors is required because the region analyzed functions as a pathway for the movement of humans and animal migrations, and thus links Central America/Mexico with the US and Canada. The One Health approach for international collaboration on veterinary and public health research is proposed to generate the knowledge base that can translate into strategies to mitigate the risk of zoonotic diseases in the US–Mexico border.

## Conflict of Interest Statement

The authors declare that the research was conducted in the absence of any commercial or financial relationships that could be construed as a potential conflict of interest.

## Supplementary Material

The Supplementary Material for this article can be found online at http://www.frontiersin.org/Journal/10.3389/fpubh.2014.00177/abstract

## Footnotes

^1^http://www.nccp.org

^2^http://www.census.gov/hhes/www/poverty/data/incpovhlth/2012/tables.html
