# Supplementary material for: Pathogenic Landscape of Transboundary Zoonotic Diseases in the Mexico–US Border Along the Rio Grande
Source: Front Public Health. 2014 Nov 17;2:177. doi: 10.3389/fpubh.2014.00177 (PMC4233934; doi:10.3389/fpubh.2014.00177)
Supplement: Supplementary file 1 [file Data_Sheet_1.DOCX]

1. **Supplemental Information**
   1. **Data and Methods for the VEEV forecasting models**

*Data Source*. Six reports and research articles provided core information on the timing, areal extent and severity of VEE in the Americas (47, 54, 67, 73, 77, 304). These authors provided a review of the location, spread, start and end dates, intensity in terms of numbers of diseased and dead equines and humans, and in specific cases, the identity of serological strains, and the application of control measures such as restriction of equine movement, vaccination, and insecticide spray programs.

*Quantifying Areal Extent*. To achieve precision, the inclusive North, Central and South American region with known VEE outbreaks was stratified into 32 smaller geographic units, typically based on state and country boundaries. The 32 geographic units with reported VEE over the century included: South America: Bolivia, Columbia, Ecuador, Peru, Trinidad, Venezuela; Central America: Costa Rica, El Salvador, Guatemala, Honduras, Nicaragua, Panama; North America: *Mexico* -- States of Campeche, Chiapas, Guanajuato, Guerrero, Hidalgo, Mexico, Michoacán, Morelos, Nayarit, Nuevo León, Oaxaca, Puebla, Querétaro, Quintana Roo, San Luis Potosi, Tabasco, Tamaulipas, Tlaxcala, Veracruz; *United States* – Texa. The fraction of the state or country affected was estimated from reports of VEE incidence in each year, and that fraction multiplied by its geographic area, obtained from several websites, notably *http://geography.about.com*, and *http://en.wikipedia.org/wiki*. These sources provided current human population levels from which we estimated population density, used as a potential predictor in our analysis of ‘drivers’ of VEE virulence.

*Quantifying Severity*. Authors reported the size of diseased populations and number of deaths for both equines and humans. Infrequently, the level of affected bovines was reported. A problem inherent in the literature reviewed was inconsistency in reporting both the number of diseased individuals and the number of deaths. The majority of reports estimated only deaths, or total numbers affected, but not both. To overcome this issue, the cases where both disease and death are reported were extracted and an average ratio of diseased**:** death cases was computed. This ratio varied depending on the magnitude of the epidemic, the ratio being much smaller in cases where the reported diseased population was small. Hence, we used one ratio for equine epidemics above a certain level (e.g., >2,000 equine diseased) and another ratio for smaller epidemics (e.g., <1000 equine diseased). Separately, the same was done for human epidemics with thresholds of >20,000 and <3000 individuals. These ratios were then applied to obtain an approximate estimate of the statistic that was lacking.

Beforehand, we could not know the logical or preferred algorithm to use as a measure of severity. For example, deaths imply greater severity than does simply the level affected (diseased). Equally, VEE in humans, and especially human deaths, imply greater severity than for equines -- given humans have access to immediate medical support. The fact that human populations are in concentrated urban areas where sanitation and drainage (as a means of mosquito control) is typical implies a VEE-affected or dead humans may signal greater severity of the disease than in equines. Hence, the arrays of location, areal extent, year, number of diseased, and number of deaths in our data matrix were tagged in such a manner that each column and value could be separately weighted using a parameter code. The concept was to test the effect of each weighting algorithm on a final index of severity. A choice could then be made on the basis of experimental outcomes.

*Integration into Single Index*. Ideally, we sought to quantify VEE incidence using one numerical scale – based on component elements. We achieved this by simply adding our measure of areal extent to severity. The latter was a combination of dead and diseased equines and humans, variously weighted. Each component index (area, human severity, equine severity) and the integrated VEE index were plotted as a time-series. Potential climate ‘drivers’ were superimposed on the integrated index and an interpretation of the impact of weather on VEE outbreak (and on its disappearance 1972-1992) was made.

*Climate Drivers of VEE*. Heavy persistent rainfall is often reported to have preceded and remain concurrent with VEE outbreaks. For example, USDA APHIS (48) reported an analysis of the very severe, high mortality (32,00 human cases with 190 deaths) viral outbreak of 1961-1964 on the Guajira Peninsula of Columbia and Venezuela -- in the 7 months preceding and the 4 months following onset of VEE, the rainfall was double that of the 5-year average for the area. To be meaningful for the large region involved, we choose to use two broad-scale ocean climate indicators -- the Accumulated Cyclonic Energy index (ACE), (*see* [*http://www.aoml.noaa.gov/hrd/tcfaq/*](http://www.aoml.noaa.gov/hrd/tcfaq/) *E11.html,* with updates for 2013 from [*http://policlimate.com/tropical/*](http://policlimate.com/tropical/) accessed May 2, 2014) that correlates with hurricane activity bringing heavy rains to countries of the Caribbean Region, and the equatorial Pacific Ocean Southern Oscillation Index (SOI), a component of ENSO (El Nino - Southern Oscillation) associated with extreme weather patterns strongly affecting the region. These climate indicators were superimposed on the VEE integrated index to determine if climate was of possible predictive value.
